# Dynamic Microenvironment Induces Phenotypic Plasticity of Esophageal Cancer Cells Under Flow

**DOI:** 10.1038/srep38221

**Published:** 2016-12-02

**Authors:** Gizem Calibasi Kocal, Sinan Güven, Kira Foygel, Aaron Goldman, Pu Chen, Shiladitya Sengupta, Ramasamy Paulmurugan, Yasemin Baskin, Utkan Demirci

**Affiliations:** 1 Bio-Acoustic MEMS in Medicine Laboratory, Canary Center at Stanford for Early Cancer Detection, Department of Radiology, Department of Electrical Engineering (by courtesy), Stanford School of Medicine, Palo Alto, California, 94304, USA; 2 Department of Basic Oncology, Institute of Oncology, Dokuz Eylul University, Izmir, 35340, Turkey; 3 Izmir International Biomedicine and Genome Institute, Dokuz Eylul University, Izmir, 35340, Turkey; 4 Department of Radiology, Molecular Imaging Program at Stanford, Bio-X Program, Stanford University School of Medicine, Palo Alto, California, 94304, USA; 5 Department of Medicine, Harvard Medical School, Boston, Massachusetts, 02115, USA

## Abstract

Cancer microenvironment is a remarkably heterogeneous composition of cellular and non-cellular components, regulated by both external and intrinsic physical and chemical stimuli. Physical alterations driven by increased proliferation of neoplastic cells and angiogenesis in the cancer microenvironment result in the exposure of the cancer cells to elevated levels of flow-based shear stress. We developed a dynamic microfluidic cell culture platform utilizing eshopagael cancer cells as model cells to investigate the phenotypic changes of cancer cells upon exposure to fluid shear stress. We report the epithelial to hybrid epithelial/mesenchymal transition as a result of decreasing E-Cadherin and increasing N-Cadherin and vimentin expressions, higher clonogenicity and ALDH positive expression of cancer cells cultured in a dynamic microfluidic chip under laminar flow compared to the static culture condition. We also sought regulation of chemotherapeutics in cancer microenvironment towards phenotypic control of cancer cells. Such *in vitro* microfluidic system could potentially be used to monitor how the interstitial fluid dynamics affect cancer microenvironment and plasticity on a simple, highly controllable and inexpensive bioengineered platform.

Cancer tissues are highly complex and heterogeneous structures, consisting of blood vessels, extracellular matrix and multiple cell types, such as cancer cells, fibroblasts, vascular, and immune cells^1^. Tumor microenvironment is not only a composition of biological and chemical regulators but also significantly affected by physical parameters such as mechanical stress and interstitial fluid flow. Changes in the physical conditions of the tumor microenvironment, driven by elevated tissue growth, proliferation of tumor cells and angiogenesis, may introduce exposure of laminar fluid flow and flow-driven shear stress on cancer tissue, which affects the level of heterogeneity and plasticity of cancer cells^2^,^3^,^4^,^5^,^6^. Bioengineering of *in vitro* cancer tissues, aiming to recapitulate the cancer microenvironment, provides powerful tools to understand the mechanisms of tumor dynamics^7^,^8^. However, conventional experimental models fail to mimic the physical cues on tumor microenvironment^9^,^10^. Revealing the role of physical dynamics that shape the behavior of cancer is key to elucidating the mechanisms underlying disease progression, and may lead to new diagnostics and therapeutic approaches^11^. Implementing bioengineering tools, such as microfluidic approaches in cancer biology, can assist to achieve novel and powerful insights in the field^7^,^9^,^10^,^12^. Microfluidic systems can provide venues to observe the effect of external stimuli of a biological system (e.g., pH, temperature, signaling factors, interstitial flow) on the *in vitro* bioengineered platforms under well-controlled miniaturized volumes and microenvironment. Such systems can be utilized to investigate the biological questions such as cell-cell and cell-material interaction, chemotherapeutic drug administration, single cell analysis, tumor metastasis. Among the efforts to mimic the physical exposures (such as the shear stress) of tumor microenvironment, diverse bioengineered platforms have been developed^13^. The effect of malignant ascites streams on ovarian cancer cells and their behavior have been earlier investigated on a microfluidic chip^14^. Designed platform is utilized to demonstrate that under continuous laminar flow and static conditions, ovarian cancer cells formed nodules, which showed significantly different metastatic profiles. Similarly, microfluidic systems have been designed to recapitulate complex transport and drug responses at the tumor microenvironment that cannot be emulated on conventional static culture models that lack the dynamics of interstitial fluid flow^15^,^16^,^17^. Many studies show the effect of the flow-induced shear stress on the vascular endothelial cells and the changes on their cellular physiology^18^. However, a limited number of studies focus on the effect of flow-mediated dynamic culture conditions on cancer cells and more investigations are needed to better understand the cancer microenvironment^19^.

To further delineate how flow-based shear stress may affect the phenotypic plasticity in terms of switching from epithelial to mesenchymal character of cancer cells, we integrated *in vitro* cell culture techniques within a dynamic laminar flow-based microfluidic platform. We chose esophageal cancer due to its highly dynamic physiologic tumor microenvironment. The esophagus is exposed to peristalsis contractions during the movement of dietary contents to the stomach, and backward flow of stomach acids in the case of gastroesophageal reflux^20^,^21^. Moreover, it is continuously subjected to shear forces through its extensive lymphatics and vascular network^22^. We herein engineered a microfluidic system to evaluate the effect of shear stress on a model system to partially represent the microenvironment of esophageal pathologies and report the effects of fluid flow on the phenotypic plasticity of these cancer cells, in effort to demonstrate the efficacy of bioengineered systems as novel *in vitro* cancer models.

## Results and Discussions

### Microfluidic platform design for dynamic cancer cell culture

**Figure 1:**
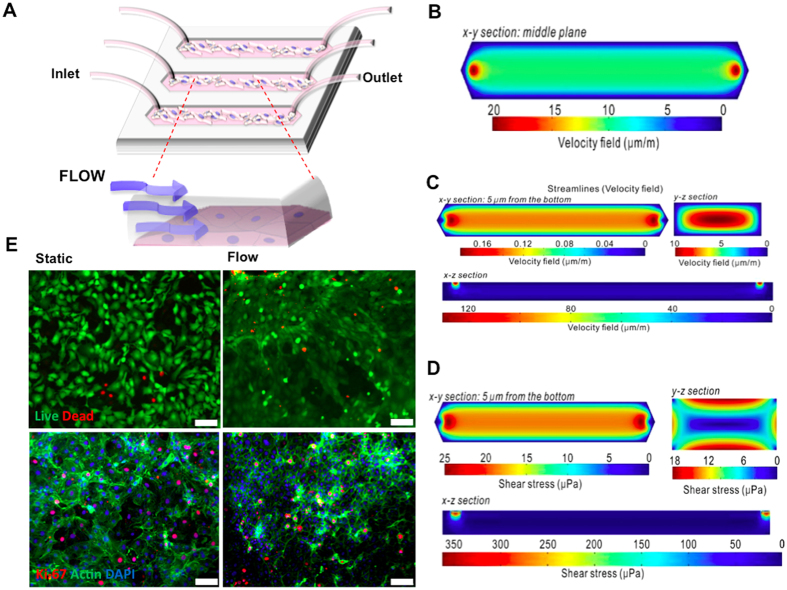
(**A**) Microfluidic platform design for dynamic cancer cell culture. (**B**) Computational fluid dynamics (CFD) simulation of velocity field at the middle layer of a microchannel showing velocity field distribution. (**C**) CFD simulation of velocity field on the XY, YZ and ZX planes demonstrating that velocity field on the surface of microchannel is uniform and around sub-micrometer per second on the bottom of the microchannel. (**D**) CFD simulation of shear stress on the XY, YZ and ZX planes indicates that shear stress distribution is uniform and around tens of micro Pascal on the bottom of the microchannel. All the simulation was performed by Comsol Multiphysics™ and plotted using pseudo-color. (**E**) Microfluidic flow does not affect the viability or proliferation of cancer cells. Cells were stained with a Live/Dead viability kit and proliferation marker Ki-67 to determine the effect of laminar microfluidic flow. Upper row, live cells are shown in green and dead cells in red color; bottom row, actin filaments (green), Ki-67 (red) (Scale bar 100 μm).

We have designed a microfluidic platform that accommodates cancer cells and optimize their sustained viability and growth. To accomplish this, we first theoretically evaluated and characterized the physical environmental parameters such as channel designs, flow rate and patterns in order to assess and predict their influences on the cells. It is critical that the cells seeded within the microfluidic channel are exposed to uniform and laminar fluid flow and thereby all feel the same physical stress through their membranes^23^,^24^. The flow in the microfluidic channel changes as a function of location. To evaluate the uniformity of fluid shear stress along the penetration path, we first derived a computational model (Fig. 1B–D, for details of the model see methods section). Reynolds number (Re) describes whether the flow within a system is laminar (Stocks flow) or turbulent according to the ratio of the inertial forces and viscosity forces. Reynolds number in the presented bioengineered platform (Re = 0.0173) belongs to Stokes flow regime for microfluidic systems (Re < 0.1). In the Stokes flow, average wall shear stress at the middle of the channel is calculated as 1.44 × 10^−5^ Pa. Mechanical microenvironmental factors such as flow-induced shear stress can modulate the physiology of cancer cells (e.g., cell cycle, metastatic character)^25^. To evaluate local wall shear stress distributions in a microfluidic channel, we performed computational fluid dynamics using finite element software, COMSOL Multiphysics^TM^. The steady state Navier-Stokes equation for incompressible Newtonian fluids was solved. The flow velocity and shear stress are illustrated in Fig. 1B–D. These results demonstrated that adherent cancer cells in the microchannels experience a simulated uniform wall shear stress of up to 25 μPa.

Esophageal adenocarcinoma cells were seeded in microfluidic channels and supplied with continuous laminar fluid flow through gas permeable tubing (Fig. 1A and Figure S1). The microfluidic system is incubated at 37 °C and 5% CO_2_ atmosphere as we reported earlier under constant flow^14^. The interstitial flow values for carcinoma has been adapted from literature^13^. Cells were exposed to 5 μm/s continuous laminar fluid flow for up to 7 days and the alterations on the plasticity related with epithelial and/or mesenchymal phenotypes of esophageal cancer were monitored by immunocytochemistry for E-cadherin, N-cadherin and vimentin. An identical system at static/no flow conditions where the culture media has been replaced manually twice a day was used as a control.

### Microfluidic platform sustains the cell viability and proliferation

Next, viability and proliferation analysis were performed to determine the effects of long-term microfluidic cell culture and to validate engineered dynamic microenvironment as a suitable niche for esophageal cancer cells. Cell viability was assessed for up to 9 days of dynamic culture and compared with static control condition using fluorescent Live/Dead assay. Cell viability in flow conditions was observed to be over 95% compared to the controls (p-value >0.05, Mann-Whitney Test) (Fig. 1E). Proliferation capacity was evaluated with the immunocytochemical expression levels of nuclear proliferation specific marker Ki-67. Both flow and control groups showed similar levels of proliferation (p > 0.05, Mann-Whitney Test) (Fig. 1E). These results further demonstrated that microfluidic platform presented here can host and sustain cancer cells within a flow-based dynamic culture model.

### Switch between epithelial and mesenchymal phenotypes under microfluidic flow

**Figure 2:**
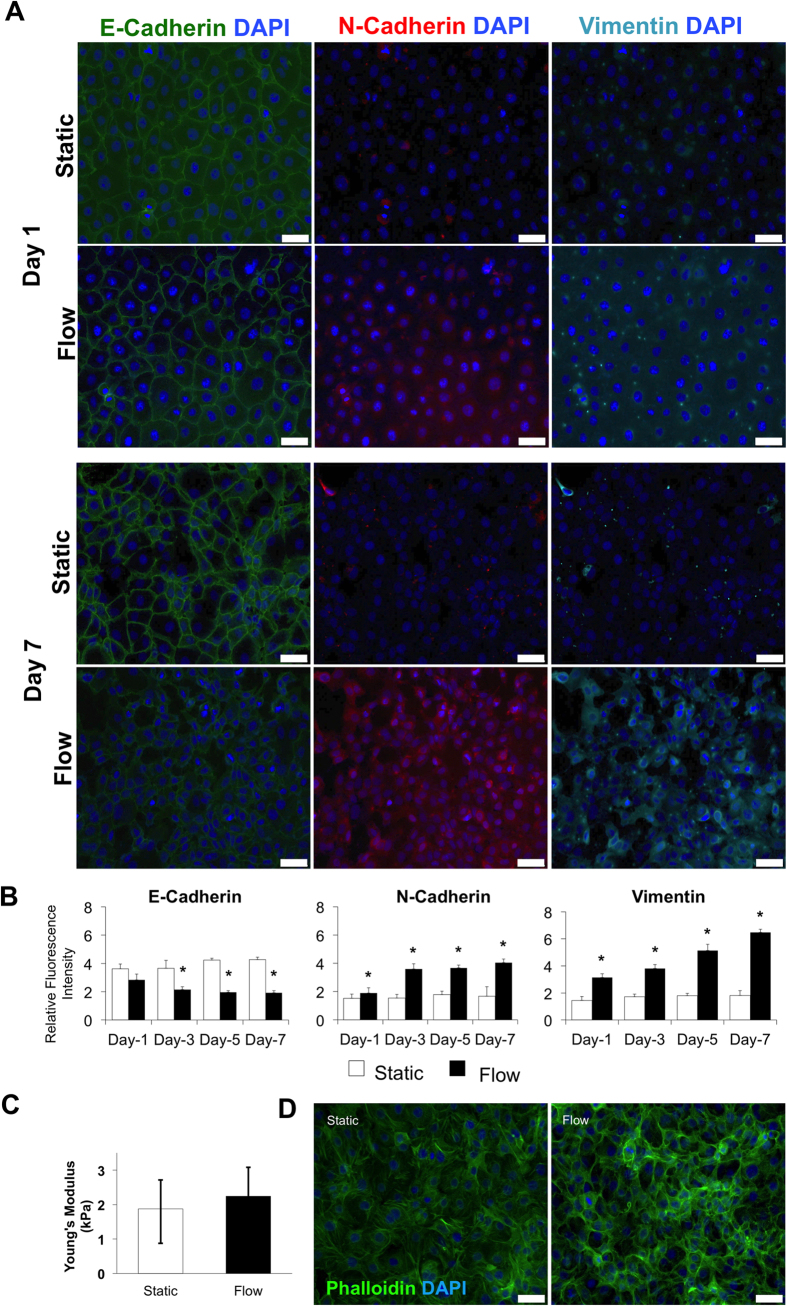
Comparative analysis of epithelial and mesenchymal markers expressions. (**A**) Fluorescence immunocytochemistry staining for E-cadherin (green), N-cadherin (red) and vimentin (cyan) was performed on esophageal cancer cells under static and microfluidic flow conditions at different time points. (**B**) Bar graphs demonstrating the intensity of expressed E-cadherin, N-cadherin and vimentin under flow and static conditions on days 1, 3, 5, 7. Quantification of fluorsence intensities from arbitrary images of each condition was done with ImageJ software (NIH, Bethesda, MD, USA). The intensities were calculated as mean ± SE (n ≥ 5), star (*) indicates p < 0.05. Immunocytochemical analysis showed that fluid flow caused 21.6% decrease in E-cadherin expression at day 1 and 54.8% decrease at day 7 compared to the static control conditions (Fig. 2A,B). In parallel, we observed flow mediated 1.24-times increase in N-cadherin expression at day 1 and 2.41-times increase at day 7 compared to control conditions (Fig. 2A,B). Furthermore, we confirmed the gain of mesenchymal features under fluidic flow induced shear stress by detection of the intracellular expression of vimentin. At day 1, vimentin expression under flow conditions was 2.15-times higher than the static controls and this increase continued to a 3.54-times at day 7 (Fig. 2A,B). To define the changes in experimental groups, the ratio of flow and static mean fluorescence intensity values of static samples were used. (**C**) Atomic force microscopy (AFM) was used to measure the cell stiffness. Bar graphs demonstrating the Young’s Modulus of cells under static control and flow conditions. (**D**) Esophageal cancer cells were stained with Phalloidin to visualize the actin microfilaments, and with 4′, 6-diamidino-2-phenylindole (DAPI) to visualize nuclei under static and laminar microfluidic flow conditions. (Scale bars: A and C, 50 μm). All data were expressed as mean ± S.E.M., n = 3, star (*) indicates, p < 0.05.

Stochastic switching of phenotypic states within populations of cancer cells can contribute to malignant features, including chemoresistance and metastasis^14^,^26^. Metastatic cells, which lose cell-cell adhesion, tend to gain tendency to enter the vascular or lymphatic circulation through epithelial to mesenchymal transition (EMT)^26^. Therefore, we examined how shear flow affects the phenotype of cancer cells by evaluating markers of both epithelial and mesenchymal states. For example, acquisition of a motile mesenchymal phenotype needed for the migration of cancer cells occurs through decrease in E-cadherin, elevation of N-cadherin and vimentin expressions^27^. Esophageal adenocarcinoma cells were exposed to continuous laminar flow for a period of 7 days in microchannels prior to immunocytochemical analysis of mesenchymal markers (E-cadherin, N-cadherin and vimentin) on days 1, 3, 5 and 7. Cancer cells under flow condition demonstrated a statistically significant decrease in cell-cell adhesion mediated by a decrease in E-cadherin expression and an increase of transendothelial migration capacity through elevation of N-cadherin expression. The phenotypic switch could be observed from the third day of culture under fluid flow (Fig. 2A,B). Immunocytochemical analysis showed that under flow conditions there is 21.6% decrease in E-cadherin expression at day 1 and 54.8% decrease at day 7 compared to the static control conditions (Fig. 2A,B).

In parallel, we observed flow mediated 1.24 -times increase in N-cadherin expression at day 1 and 2.41-times increase at day 7 compared to static control conditions (Fig. 2A,B). Furthermore, we confirmed the gain of mesenchymal features under fluidic flow induced shear stress by detection of the intracellular expression of vimentin. At day 1, vimentin expression under flow conditions was 2.15-times higher than the static controls and this increase continued to a 3.54-times change at day 7 (Fig. 2A,B). Intriguingly, under laminar fluid shear stress esophageal adenocarcinoma cells present a hybrid epithelial/mesenchymal (E/M) state without any EMT induction through cytokines such as TGF-β. Gaining a hybrid epithelial-mesenchymal (E/M) state, which includes both epithelial and mesenchymal phenotype (epithelial E-cadherin is expressed simultaneously with mesenchymal markers N-cadherin and vimentin), can occur during the switch between the epithelial to mesenchymal transition and the mesenchymal to epithelial transition^28^,^29^. Indeed, the hybrid, E/M phenotype is associated with malignant features such as an acquired drug resistant state^30^. Our results indicate that fluid flow triggers the EMT in the absence of any type of chemical or biological stimuli within the bioengineered microenvironment.

### Cellular morphology and stiffness changes upon fluid shear stress

Physical microenvironmental stimuli can dramatically influence the cellular metabolism and cell membrane properties such as cell stiffness^31^,^32^. We evaluated the physical effects of shear stress on cell membrane and cell morphology. The cell membrane of cancer cells cultured under flow for 7 days was analyzed with atomic force microscopy (AFM). Importantly, to avoid bias due to the fixative driven stiffness, AFM analysis was performed on viable cells. We observed that cells exposed to 7 days of flow showed an increase in cellular stiffness compared to a static condition (Fig. 2C). The membrane stiffness for flow-conditioned cells was measured as 2.24 ± 1.02 kPa and for static controls as 1.87 ± 1.02 kPa. The cell stiffness is often associated with the remodeling of the actin cytoskeleton^31^,^33^. Actin filament network in the cellular organization, determines the shape, stiffness and cell motility as well as plays a crucial role in transduction of mechanical signals, muscle contraction, cell division, invasion and EMT^34^,^35^,^36^. The presence of stressed F-actin fibers and microtubules may induce fibroblast-like changes in cell shape during EMT^37^,^38^. We used fluorescently labeled actin binding marker Phalloidin to visualize stressed actin filaments in select subcellular locations and fibroblast-like changes in cell morphology after 7 days of cell culture under flow conditions (Fig. 2D). The switch between epithelial and mesenchymal states of cancer cells, is not only a biological process at a molecular level but also involves physical and mechanical regulations at each step of the cascade. Our data from cancer cells exposed only to flow-based shear stress is in agreement with the previously reported correlation between biochemical and physical changes at phenotype transformation phase^17^. Changes in actin cytoskeletal network of esophageal adenocarcinoma cells represent the alteration of expressions profile of both epithelial and mesenchymal markers. These results indicate that flow-based shear stresses on cancer cells may allow the existence of a hybrid epithelial/mesenchymal phenotype (E/M)^39^,^40^.

### Dynamic microenvironment promotes stem cell like character of esophagus cancer cells

**Figure 3:**
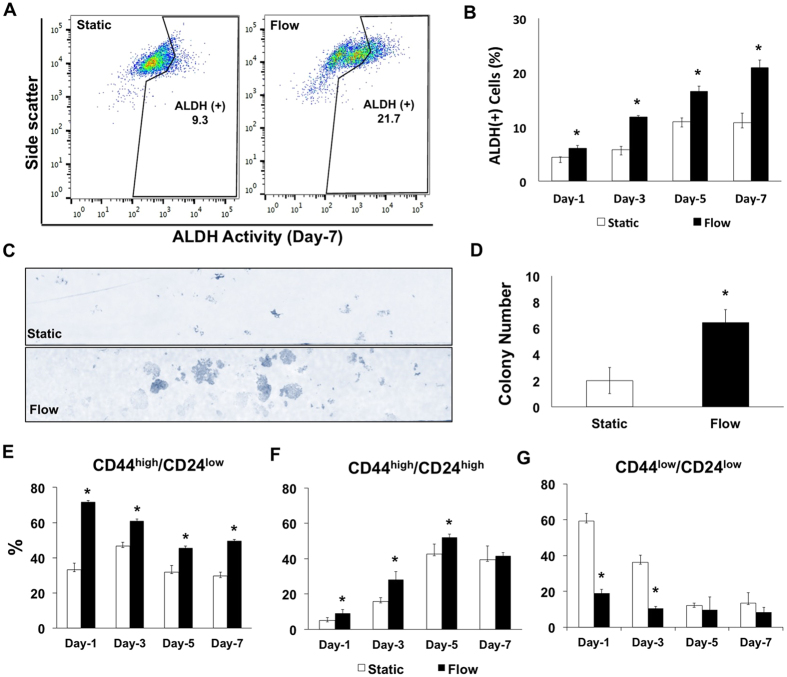
(**A**) The percentage of ALDH-positive esophageal cancer cells increase under laminar flow in the microchannel. FACS analysis of ALDH expression of cancer cells under static and flow conditions on day 7. (**B**) ALDH-positivity percentages of esophageal cancer cells under static and flow conditions on day 1, 3, 5 and 7. (**C**) Microscopic images of colony forming assay under static and flow conditions. (**D**) Bar graphs demonstrate the colony number under static and flow conditions. The colony forming capacity of esophageal cancer cells increases under laminar microfluidic flow (*p ≤ 0.05). (**E**, **F**, **G**) FACS analysis of CD24 and CD44 expression at different time points of esophageal cancer cells under static and laminar flow conditions (*p ≤ 0.05). All data were expressed as mean ± S.E.M., n = 3, star (*) indicates, p < 0.05.

Drug detoxifying enzyme aldehyde dehydrogenase (ALDH) positivity, as a poor prognostic factor, is identified in lung, prostate, breast, colon and esophageal cancers and has been linked to mesenchymal fate of cells and EMT^41^,^42^. We observed a statistically significant shift in ALDH-positive population once cultured under laminar shear stress and their ALDH level has been monitored up to 7 days (Fig. 3A,B and Figure S2). Under flow conditions the ALDH-positive population increases from 6.0% to 20.9%, whereas the increase under static controls remains from 4.4% to 10.8%. ALDH-positive cells have greater colony forming ability, which can be associated with higher metastatic progression and higher number of tumor-initiating cells^43^,^44^. Indeed, the colony formation assay under flow conditions demonstrated 2.2-fold higher capacity than the static controls (Fig. 3C,D). Cancer cells cultured under static conditions express ALDH^45^,^46^. In our experimental design, both cancer cells in static and flow conditions proliferate (thereby cell number increases) over 7 days of culture period. The culture conditions such as seeding density and duration is attributed to change the physiology of cells *in vitro*^47^. The increase in ALDH-positive cells under static condition is attributed to the increased cell-cell contact over the time course of the experiment. The proportion of ALDH-positive cancer cells changes with cell culture density due to the expression of different ALDH isoforms^48^. When static and flow conditions were compared to each other at time points (days 1, 3, 5 and 7) only the ALDH-positive increase in flow mediated cells has been observed as significant. The physical fluid shear stress induces the elevation of ALDH-positive cell fraction together with hybrid E/M state, suggesting a shift towards aggressive and cancer stem cell-like phenotype^49^.

Further, we investigated the effect of physical stimulation through flow-based shear stress on well-studied cancer cell populations. CD44, a multifunctional class I transmembrane glycoprotein, and CD24, a small cell surface protein, are widely expressed in various cancer cell populations, and are described as cancer stem-like cells capable of initiating invasion, metastasis, therapeutic resistance, heterogeneity and plasticity in tumors^30^,^50^,^51^,^52^. Our results demonstrate that CD44^low^/CD24^low^ cells show phenotype switching to CD44^high^/CD24^low^ and CD44^high^/CD24^high^ under both static and flow conditions towards day 7 (Fig. 3E–G and Figure S3). Cell surface markers are dynamically expressed, even under static conditions, as a result of active signaling mechanisms and microenvironmental conditions. A decrease in the expression of CD44^low^/CD24^low^ cells was statistically significant on the first and third day of culture under flow when compared with the results under static condition. The percentages of CD44^high^/CD24^low^ (mesenchymal-like; M) and CD44^high^/CD24^high^ (hybrid phenotype; EM) cell populations under flow conditions were significantly higher than under static conditions. Fluid flow could be a driver of phenotypic switching into the EM and M phenotypic states. These states are shown as one of the main reasons that cancer therapy is often unsuccessful^2^,^30^,^53^. In *in vitro* esophageal cancer studies, a CD44^high^/CD24^low^ sub-population predicts a reduced response to chemotherapy, radiation and a high capacity for metastasis, e.g. with breast cancer, because of the increased aggressive behavior of the tumor^54^,^55^. Recent evidence now suggests that CD44^High^/CD24^High^ (M) phenotype, between epithelial and mesenchymal states, can be significantly more metastatic and indicative of an adaptive resistance to chemotherapy^30^. Based on these reports, it is possible to gain insights into CD44 and CD24 expression and their possible roles in metastasis and resistance to cancer therapies. However, no studies have been reported on the status of these markers under flow conditions^54^,^55^,^56^.

In Fig. 3E–G we monitor how flow changes the CD24 and CD44 expressions on esophagus cancer cells. JH-EsoAd1 esophagus cancer cell line is a heterogeneous cell line, which consists of multiple sub-populations, and has been extensively characterized elsewhere^57^,^58^,^59^. The *in vitro* culture course of the cells leads alterations on their physiology and cell surface markers such as CD24 and CD44. In this study, we have analyzed the CD44^high^CD24^low^, CD44^high^CD24^high^ and CD44^low^CD24^low^ populations under flow and static conditions. The CD44^high^CD24^low^ sub-population has been attributed to more tumorogenic and cancer stem cell character as well as exhibiting EMT characteristics^60^,^61^. Our results in Fig. 3E shows that under flow conditions CD44^high^CD24^low^ population is significantly higher than the static condition for 1 week of culture period. This observation correlates with findings in Fig. 2A,B as flow induces the EMT. Similarly to slight increase in ALDH expression under static conditions the shift from CD24^low^ to CD24^high^ in static conditions can be attributed to elevation of cell density within the confined environment over time^62^,^63^.

### Microfluidic cancer cell culture platform as test bed for chemotherapeutic administration

**Figure 4:**
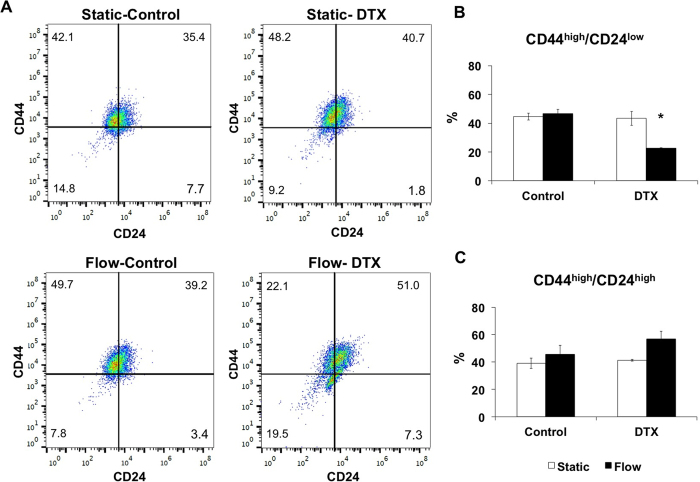
(**A**) Representative plots showing the distribution of CD44^high^/CD24^low^ and CD44^high^/CD24^high^ cells for control and docetaxel (3 nM) groups under static and laminar microfluidic continuous flow. (**B**) Bar graphs demonstrating the percentages of CD44^high^/CD24^low^ cells in static and laminar microfluidic flow conditions w/and w/o docetaxel. (**C**) Bar graphs demonstrating the percentages of CD44^high^/CD24^high^ cells in static and laminar microfluidic flow conditions w/and w/o docetaxel. All data were expressed as mean ± S.E.M., n = 3, star (*) indicates, p < 0.05.

The presented microfluidic-based dynamic cancer microenvironment can be potentially utilized in chemotherapeutic administration studies. The phenotypic plasticity and metastasis capacity can be altered by the interactions between cancer cells and dynamic microenvironment. We evaluated the response of esophageal cancer cells to a chemotherapeutic agent (e.g., docetaxel) in well-defined and controlled administrative regimes. Docetaxel (DTX) is used as a second line therapy in aggressive cases of esophageal adenocarcinoma and has been successfully evaluated in the clinic for over a decade. Miyanaga *et al*. have reported that docetaxel is not cytotoxic for low concentrations (<10 nM) and enhances cell death in concentration dependent manner^64^. We applied 3 nM DTX in our microfluidic platform to observe the chemotherapeutic response of cancer cells, in particular to evaluate the CD44 and CD24 expression. Continuous delivery of DTX decreases the expression of CD44^high^/CD24^low^ population from 46.54 ± 3.1% to 22.58 ± 0.4% and increases CD44^high^/CD24^high^ populations from 45.6 ± 6.4% to 56.8 ± 5.8% under flow (Fig. 4A–C). Under DTX administration when the fluid flow is applied CD44^low^ population is increasing from 7.8 ± 3.2% to 18.1 ± 8.7%. We observe that CD24 expression at flow conditions increases both in combination with DTX administration and its absence. The correlation of the increase in CD24 expression with the increase of malignancy of the cancer cells has been previously reported^52^. During disease progression, cancer cells are able to demonstrate adaptation and phenotypic changes such as escape from apoptosis, resistance to chemotherapeutics and increase in motility, invasiveness and migration, which are triggered by the transition between epithelial and mesenchymal phenotype^49^,^53^. Flow-based phenotypic plasticity we observe here can be a potential mechanism for enabling cells to survive and adapt to environmental changes.

Understanding the nature of cancer cells in their native dynamic microenvironment, in terms of physical and physiological components, is essential^65^,^66^. Intrinsic mechanical forces such as interstitial fluid flow-based shear stress might be one of the reasons for inconsistencies between *in vitro* experimental and clinical observations. Bioengineered *in vitro* cancer microenvironments and model platforms could potentially provide more reliable information than traditional *in vitro* static culture systems. This study demonstrates how flow can direct the phenotypic plasticity in esophageal cancer cells in terms of modulating the EMT based molecular expression signatures, clonogenic potential, ALDH, and CD44/CD24 expressions. The presented platform can be further utilized in cancer research for understanding transcriptomic and genomic variations (e.g., signaling mechanisms, cellular heterogeneity, mutations) and controlling physiochemical parameters (e.g., pH and hypoxia level, matrix stiffness). Investigating flow-based plasticity and its effects on cell fate, through microfluidic model platforms will potentially further accelerate the progress of cancer management and pharmaceutical applications. In particular, with advances in microfluidic cancer cell culture techniques, developing new personalized therapy strategies investigating the unknowns in tumor heterogeneity and chemotherapeutic resistance are interesting for future research in cancer.

## Methods

### Cell culture

The esophageal adenocarcinoma cell line, JH-EsoAd1^57^, a kind gift from Dr. James R. Eshleman (The Johns Hopkins University, Baltimore, Maryland, USA), which was previously characterized by Dr.Eshleman, was maintained in complete culture medium containing high-glucose Dulbecco’s Modified Eagle Medium (DMEM with 4 g/L glucose, 4.0 mM L-Glutamine) (Gibco by Life Technologies, Grand Island, NY, USA) supplemented with 10% heat-inactivated FBS (Gibco Life Technologies, Grand Island, NY, USA), and 1% Penicillin-Streptomycin (Gibco by Life Technologies, Grand Island, NY, USA) at 37 °C with 5% CO_2_ in microfluidic chips. In the static culture group, the media was changed manually two times per day to minimize the potential effects of hypoxia. In the flow group, adherent cells were continuously supplied with fresh medium using a *programmable syringe pump (NewEra Pump Systems Inc., NY) at* a flow rate of 2 μl/min. The aldehyde dehydrogenase (ALDH), CD44, CD24 measurements and epithelial to mesenchymal marker stainings were performed on days 1, 3, 5 and 7 to determine the phenotypic plasticity under flow and static conditions.

### Design and fabrication of the microfluidic chips

The microfluidic chips were fabricated using 1.5 and 3.175-mm-thick polymethyl methacrylate (PMMA) (McMaster Carr, Santa Fe Springs, CA, USA), 50 μm thick double-sided adhesive (DSA) film (Saunders, MN, USA) and polystyrene petri dishes (BD Falcon, Franklin Lakes, NJ, USA) (Figure S1). Polystyrene layers from petri dishes were used for the bottom of the chips to achieve effective cell attachment. Outer dimensions of microfluidic chips components were cut to 25 × 41 mm dimensions (VersaLASER; Universal Laser Systems Inc., AZ, USA). Three channels (width: 4 mm, length: 27 mm) were cut from 1.5 mm thick PMMA plates to support the cells with cell culture media in the channels. The channel length is referred to as the distance between the inlet and outlet ports. The polystyrene bottom layer and a 1.5 mm thick PMMA layer (with channels) were bound by a DSA film. The upper part of the chip, with inlet and outlet ports, was cut from 3.175 mm thick PMMA sheets. The diameter of the inlet and outlet ports was 0.7 mm. All components of the microfluidic chip were sterilized with 70% ethanol followed with ultraviolet light exposure under laminar flow hood for an hour. Entire assembly process was performed under aseptic conditions. Before connecting the silicon-based tubing to the inlet/outlet ports, a suspension of JH-EsoAd1 cells was manually inoculated into the channels of the microfluidic chip.

### Cell seeding in microfluidic chips

Cells at 5.58 × 10^4 ^cells/channel seeding density were loaded in each microfluidic channel and incubated for 24 hours to achieve cell adhesion and homeostasis. Gas-permeable silicon tubing 0.010” inner diameter × 0.030” outer diameter (Tygon, ColeParmer, USA) was attached to the inlet and outlet ports of the microfluidic chips using epoxy glue (Devcon, Danvers, MA, USA). Syringes containing cell culture medium were connected to the tubing. For the static condition, the culture medium was replaced twice per day by manual pipetting.

### Computational modeling of flow in the microfluidic platform Platform

Theoretical investigation of the fluid system was performed to evaluate the shear stress effect on the adherent cells. The Reynolds number in the microchannel was calculated from Equation (1):


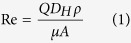


where *Q* is the volumetric flow rate (2 μL min^−1^), *D*_*H*_ is the hydraulic diameter of the pipe, *ρ* is the fluid density, *μ* is the dynamic viscosity of the fluid and *A* is the cross-sectional area of the pipe. For rectangular microchannels with comparable heights and widths, the hydraulic diameter can be expressed as Equation (2):


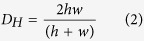


At 37 °C, the fluid density and the dynamic viscosity are 1000 kg m^−3^ and 0.000692 kg m^−1 s–1^, respectively.

The Reynold number in this microfluidic system was calculated to be 0.0173, which is in the Stokes flow regime. In Stokes flow, the average wall shear stress in the middle of channel is calculated as 1.44 × 10^−5^ Pa (or 14.4 × 10^−5 ^dynes cm^−2^), according to the Equation (3)


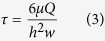


where *μ* is the dynamic viscosity of the fluid (0.000692 kg m^−1 s–1^), *Q* is the volumetric flow rate (2 μL min^−1^), *h* is the height of the microchannel (1.55 mm), and *w* is the width of the microchannel (4 mm).

The boundary layer thickness of this fluid system at the middle of the microchannel (*L*/*2*) was calculated to be 41 mm, according to the following Equation (4):


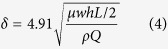


To evaluate the local wall shear stress distribution, we performed computational fluid dynamics using a finite element software, e.g. Comsol Multiphysics^TM^. The steady state Navier-Stokes equation for incompressible Newtonian fluids was solved. As the boundary layer thickness of this microfluidic system at middle of the microchannel (*L*/*2*) is larger than the height of the microchannel, the shear stress in the fluid can be expressed by Equation (5)


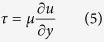


where *u* is the fluid velocity along the microchannel, and *y* is the distance from the no-slip wall at the bottom of the microchannel.

### Viability and proliferation analysis.

Cell viability was determined using a LIVE/DEAD Viability/Cytotoxicity kit (Life Technologies, USA), which provides a two-color fluorescence assay for the determination of live (green) and dead (red) cells. Both static and flow conditions were analyzed with ImageJ (NIH, Bethesda, MD, USA) for a minimum of 5 images for each condition. Proliferation of the cells was measured by staining using a mouse monoclonal antibody against human Ki-67 (Abcam, ab15580, Cambridge, MA, USA). The cells were visualized using a fluorescence microscope.

### F-actin cytoskeleton staining

F-actin cytoskeletal filaments were stained with Alexa Fluor-488 Phalloidin (Life Technologies, OR, USA) on day 7 of the microfluidic experiments to observe the morphological differences of cells under static control and microfluidic flow conditions. The cells were visualized with a fluorescence microscope.

### Fluorescence immunocytochemistry

The relative expression levels of E-cadherin, N-cadherin and vimentin were evaluated by immunofluorescence staining. Cells were fixed with 4% paraformaldehyde (Electron Microscopy Sciences, Hatfield, PA, USA), and stained with a mouse monoclonal antibody against human E-cadherin (Abcam, ab1416, Cambridge, MA, USA), a rabbit polyclonal antibody against human N-cadherin (Abcam, ab12221, Cambridge, MA, USA) and chicken polyclonal antibody against human vimentin (Novus Biological, NB300-223, Littleton, CO, USA). The cells were then incubated with the appropriate secondary antibodies: Alexa Fluor- 488 goat anti-mouse (Life Technologies, A-10667, Carlsbad, CA, *USA*), Alexa Fluor- 568 goat anti-rabbit (Life Technologies, A-11011, Carlsbad, CA, *USA*), or Alexa Fluor- 647 donkey anti-chicken (Jackson ImmunoResearch Lab, 703-606-155, West Grove, PA, *USA)*. Cells stained with E-cadherin, N-cadherin and vimentin were visualized under fluorescence microscope (Zeiss, Axio Observer, USA). Fluorescence intensity analyses of images from flow and static condition were performed with ImageJ software (NIH, Bethesda, MD, USA) (http://imagej.nih.gov/ij/), n ≥ 5. The intensity values were expressed in mean ± Standard error of for fluorescence intensity covered. The obtained mean value of fluorescence intensity was used to compare static and dynamic experimental groups^67^,^68^. To define the changes in experimental groups, the ratio of flow and static mean fluorescence intensity values of static samples were used.

### ALDEFLUOR assay

Aldehyde dehydrogenase (ALDH) activity was determined using an ALDEFLUOR Kit (Stem Cell Technologies, Vancouver, Canada) according to the manufacturer’s instructions. Cells were suspended in ALDEFLUOR assay buffer containing ALDH substrate (boron dipyrromethene aminoacetaldehyde (BAAA)) and incubated for 45 minutes at 37 °C. As a negative control, an aliquot from each sample was treated with an ALDH–specific inhibitor (diethylaminobenzaldehyde (DEAB)). Stained cells were resuspended in ALDEFLUOR assay buffer and analyzed using a BD FACSAria flow cytometry (BD Biosciences, San Jose, CA, USA).

### Colony forming unit assay

Colony forming assays were performed to evaluate the colonogenecity of the cell population under flow and static control conditions. Three hundred viable esophageal cancer cells were seeded into each channel of the microfluidic chips. On the day 4, silicon tubing was connected to the flow chips. After 3 weeks of incubation, the colonies were fixed with 4% PFA and stained with 0.01% crystal violet (Sigma Aldrich, St. Louis, MO). The colonies were counted using a microscope.

### Flow cytometry (FACS)

Trypsinized cells were incubated with anti-human CD44 antibodies (allophycocyanin) (BD Pharmingen, APC Mouse Anti-Human CD44, 559942, USA), anti-human CD24 antibodies (phycoerythrin) (BD Pharmingen, and PE Mouse anti-human CD24, 555428, USA) on days 1, 3, 5 and 7. Next, cells were resuspended in FACS buffer (1% bovine serum albumin (Sigma-Aldrich, St. Louis, MO, USA) in PBS) and then aquired using a FACSAria flow cytometer (Becton Dickinson, San Jose, California, USA). Results were analyzed with FlowJo Data Analysis Software (FlowJo LLC, OR, USA).

### Atomic force microscopy

Cell stiffness was determined with atomic force microscopy (AFM) Park XE-100 and analyzed with SPIP software. After 7 days cells cultured in static and flow conditions samples with live cells were analyzed with MLCT probes (Bruker Inc.) in liquid environment.

### Statistics

All experiments in this study were performed at least three times unless other specified. All graphs and numerical values in the figures are presented as the mean ± the *standard error* of the mean (SEM). *A* non-parametric Mann Whitney U test was used to determine the significance of the differences between flow and static samples. Repeated measure ANOVA was used to analyze the trend of differences among different time points. Data were analyzed using SPSS V.21.0 for Windows (SPSS, Chicago, Illinois, USA). The level of statistical significance was set at p ≤ 0.05.

## Additional Information

**How to cite this article**: Calibasi Kocal, G. C. *et al*. Dynamic Microenvironment Induces Phenotypic Plasticity of Esophageal Cancer Cells Under Flow. *Sci. Rep.* **6**, 38221; doi: 10.1038/srep38221 (2016).

**Publisher's note:** Springer Nature remains neutral with regard to jurisdictional claims in published maps and institutional affiliations.

## Supplementary Material

Supplementary Information
