# Supplementary material for: Dynamic Microenvironment Induces Phenotypic Plasticity of Esophageal Cancer Cells Under Flow
Source: Sci Rep. 2016 Dec 2;6:38221. doi: 10.1038/srep38221 (PMC5133540; doi:10.1038/srep38221)
Supplement: Supplementary Information [file srep38221-s1.pdf]

Dynamic Microenvironment Induces Phenotypic Plasticity of Esophageal Cancer Cells Under Flow

Gizem Calibasi Kocal[1,2,+], *Sinan Güven*[1,3+], *Kira Foygel*[4], *Aaron Goldman*[5], *Pu Chen*[1], *Shiladitya Sengupta*[5], *Ramasamy Paulmurugan*[4], *Yasemin Baskin*[2], *Utkan Demirci*[1,*]

[1]Bio-Acoustic MEMS in Medicine Laboratory, Canary Center at Stanford for Early Cancer Detection, Department of Radiology, Department of Electrical Engineering (by courtesy), Stanford School of Medicine, Palo Alto, California, 94304, USA

[2] Department of Basic Oncology, Institute of Oncology, Dokuz Eylul University, Izmir, 35340, Turkey.

[3] Izmir International Biomedicine and Genome Institute, Dokuz Eylul University, Izmir, 35340, Turkey.

[4]Department of Radiology, Molecular Imaging Program at Stanford, Bio-X Program, Stanford University School of Medicine, Palo Alto, California, 94304, USA.

[5]Department of Medicine, Harvard Medical School, Boston, Massachusetts, 02115, USA.

[+] These authors contributed equally.

* Corresponding Author: Utkan Demirci, PhD

Bio-Acoustic MEMS in Medicine Laboratory, Canary Center at Stanford for Early Cancer Detection, Department of Radiology, Stanford School of Medicine, Palo Alto, California, 94304, USA

E-mail: utkan@stanford.edu

Supplemental Figures and Legends

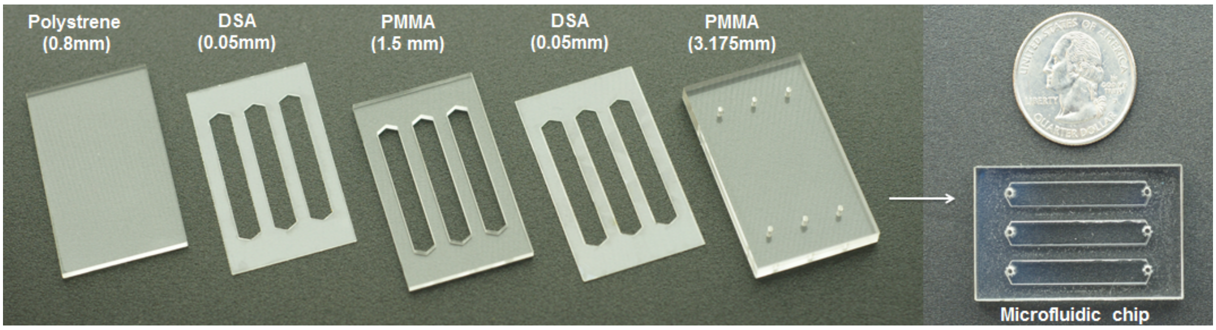

Figure S1. Components of the microfluidic chips were cut to 25 × 41 mm dimensions by using 1.5 and 3.175-mm-thick polymethyl methacrylate (PMMA), 50 µm thick double-sided adhesive (DSA) film and polystyrene petri dishes. Three channels (Width: 4 mm, length: 27 mm) were cut from 1.5 mm thick PMMA sheets to support the cells with adequate media.

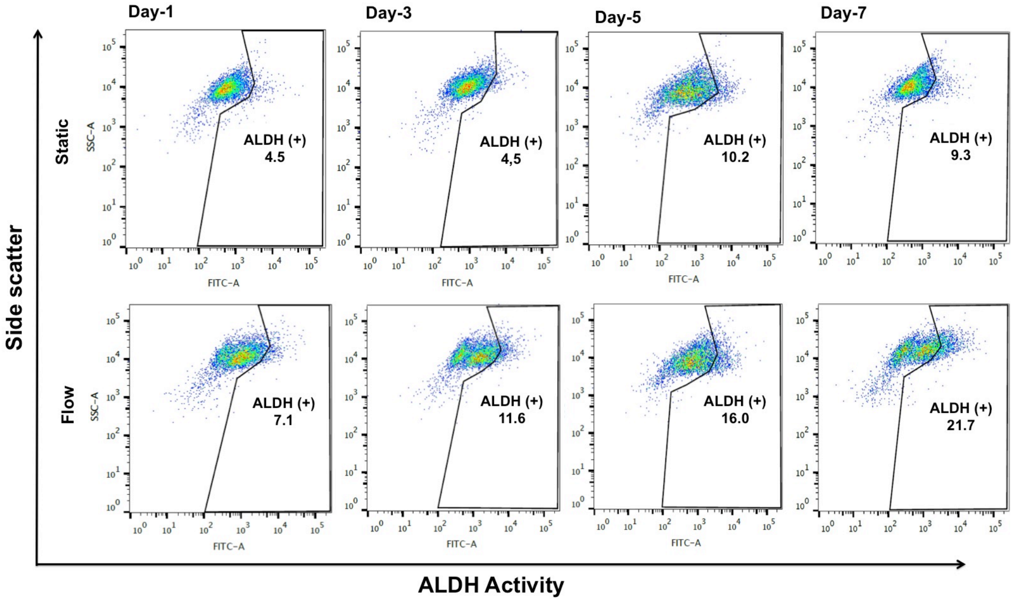

Figure S2. Drug detoxifying enzyme aldehyde dehydrogenase (ALDH) activity was assessed by using flow cytometry from static and flow conditions on days 1, 3, 5 and 7.

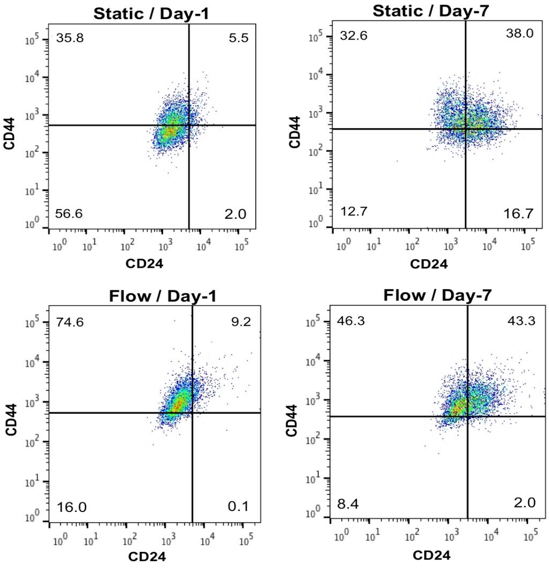

Figure S3. Flow cytometry analysis of CD24 and CD44 at different time points (Day 1 and 7) in JHesoAD1 cells under static and laminar microfluidic flow conditions.
